# The Effect of Fucoidan from the Brown Alga *Fucus evanescence* on the Activity of α-*N*-Acetylgalactosaminidase of Human Colon Carcinoma Cells

**DOI:** 10.3390/md16050155

**Published:** 2018-05-10

**Authors:** Irina Bakunina, Oksana Chadova, Olesya Malyarenko, Svetlana Ermakova

**Affiliations:** 1 Laboratory of Enzyme Chemistry of G.B. Elyakov Pacific Institute of Bioorganic Chemistry, Far Eastern Branch, Russian Academy of Sciences, Vladivostok 690022, Russia; vishchuk@mail.ru (O.M.); swetlana_e@mail.ru (S.E.); 2 School of Natural Sciences, Far Eastern Federal University, Vladivostok 690091, Russia; chadova_9595@mail.ru

**Keywords:** brown alga, *Fucus evanescence*, fucoidan, α-*N*-acetylgalactosaminidase, alpha-NaGalase, colon carcinoma cells, DLD-1, macrophage activating factor

## Abstract

α-*N*-acetylgalactosaminidase (EC 3.2.1.49) (alpha-NaGalase) catalyzes the hydrolysis of *N*-acetamido-2-deoxy-α-d-galactoside residues from non-reducing ends of various complex carbohydrates and glycoconjugates. It is known that human cancer cells express an alpha-NaGalase, which accumulates in the blood plasma of patients. The enzyme deglycosylates the Gc protein-derived macrophage activating factor (GcMAF) and inhibits macrophage activity acting as an immunosuppressor. The high specific activity 0.033 ± 0.002 μmol mg^−1^ min^−1^ of the enzyme was found in human colon carcinoma cells DLD-1. The alpha-NaGalase of DLD-1 cells was isolated and biochemical characterized. The enzyme exhibits maximum activity at pH 5.2 and temperature 55 °C. The *K*_m_ is 2.15 mM, *V*_max_–0.021 μmol min^−1^ mL^−1^, *k*_cat_–1.55 min^−1^ and *k*_cat_/*K*_m_–0.72 min^−1^ mM^−1^ at 37 °C, pH 5.2. The effects of fucoidan from the brown alga *Fucus evanescence* on the activity of alpha-NaGalase in human colon carcinoma DLD-1 cells and on the biosynthesis of this enzyme were investigated. It was shown that fucoidan did not inhibit free alpha-NaGalase, however, it reduced the expression of the enzyme in the DLD-1 cells at IC_50_ 73 ± 4 μg mL^−1^.

## 1. Introduction

According to the statistics of the World Health Organization, the colon is the second organ after lung in the human body, which is most often affected by cancer. Annually in the world about 600 thousand cases of colon cancer are fixed. Moreover, colon cancer is one of the most common cancers with a high propensity to metastasize; 30–40% of patients have metastatic disease at the initial diagnosis [1]. That’s why the improvement of colon cancer therapy is crucial task of oncology.

α-*N*-Acetylgalactosaminidases (alpha-NaGalase) (EC 3.2.1.49) are exo-glycosidases. They catalyze the hydrolysis of the terminal α-linked *N*-acetylgalactosamine residues from the non-reducing ends of various complex carbohydrates and glycoconjugates. Glycolipids, glycopeptides, and glycoproteins, blood group A erythrocyte antigens [2,3,4], lipopolysaccharides of the cell walls and capsules of bacteria [5,6,7] are physiological substrates for alpha-NaGalase.

These enzymes were found in the organs and tissues of human [8] and terrestrial mammals [9], birds [10], invertebrates [11,12], and reddish worms [13]. To date, alpha-NaGalases were revealed in the anaerobic terrestrial fungi [14,15], bacterial human pathogens of *Firmicutes* [16,17] and *Bacteroidetes* phylum [18]. In the marine environment, alpha-NaGalases have been isolated from the liver and digestive organs of the marine invertebrate [12,19,20], fish [21] and in marine bacteria of the genus *Arenibacter* [22,23,24]. Interesting to note the enzymes are not found in plants.

The enzymes take part in the catabolism of complex oligosaccharides. It was found that the deficiency of lysosomal alpha-NaGalase in the human body causes a dangerous hereditary Schindler/Kanzaki disease, which stimulated an intensive, comprehensive study of this enzyme [25]. alpha-NaGalase activity is an enzymatic basis for the fusion process and play dual roles in viral infectivity of influenza and human immunodeficiency Type I virus, as well as in immunosuppression [26,27].

According to the modern classification of carbohydrate active enzymes (CAZy), alpha-NaGalases occur in the GH27 family, while the alpha-NaGalases of bacteria take place in the related GH36 family. GH109 and GH129 families include exclusively the alpha-NaGalases of bacteria [28]. Structures and mechanisms of action of enzymes from different GH families are significantly different.

As a rule, the immune response results from the activation of macrophages. The major macrophage activation cascade involves participation of beta-galactosidase and sialidase of B and T lymphocytes, respectively, and serum vitamin D_3_-binding protein (Gc-protein). As results, macrophage activating factor (MAF) is formed. This protein with *N*-acetylgalactosamine as the remaining sugar moiety is a precursor of GcMAF-mediated macrophage activation cascade [29].

Alpha-NaGalase is universally detected in a blood plasma of a variety of cancer patients, but not in healthy individuals [30]. Previously it was established that alpha-NaGalase produced by human cancer cells and accumulated in the blood plasma was responsible for deglycosylation of Gc-protein leading to immunosuppression in advanced cancer patients [31,32,33]. Mohamad S.B. and coworkers had studied the biochemical characterization of alpha-NaGalase from several human tumor cell lines and showed its involvement in decreasing of the potency of GcMAF on macrophage activation [34,35]. The ability of alpha-NaGalase to inhibit macrophage activity in patients with developing tumors, acting as an immunosuppressor is of a great interest. Compounds inhibiting the activity of this enzyme could serve as a base for creation of an immunomodulating drugs.

The anticancer mechanism of fucoidan remains unclear. The fucoidan is sulfated polysaccharide from brown algae with wide range of biological activities including immunomodulating activity. The immunomodulatory effect is one of the possible mechanisms of the protective effect of fucoidan against cancer. The immunomodulating activity of fucoidans was described either on cellular or on humoral levels. In vivo it was shown that fucoidan was able to increase the activity of natural killer (NK) cells, which are contributed to tumor regression [36,37]. Cytotoxic T lymphocytes (CTL) are known to play a major role in the adaptive cellular immune. The fucoidan was reported to increase the amount of CTL [38]. Moreover, fucoidan was found to effectively stimulate the CTL cells associated with the dendritic cell (DC) resulting high specific lysis of breast cancer cells [39]. In vivo study revealed that fucoidan increased the production of IL-6 and IL-12 as well as TNF-α [40]. It was shown earlier that fucoidans from *Fucus vesiculosus*, *Ascophyllum nodosum*, *Sargassum wightii* effectively inhibited in vitro human pancreatic α-amylase and α-glucosidase, a key enzyme responsible for digestion and absorption of glucose in the small intestine [41,42,43]. Action of these unique polysaccharides from brown algae on cancer alpha-NaGalase previously was not investigated.

The study aimed to characterize the biochemical properties of alpha-NaGalase of human DLD-1 intestinal carcinoma and to estimate the effect of fucoidan on the activity of the free enzyme and on expression of the enzyme by cancer cells.

## 2. Results and Discussion

In the present study several human cancer cell lines such as human melanoma SK-MEL-28 and RPMI-7951, breast cancer T-47D and MDA-MB-231, and colon cancer DLD-1 and HT-29 were screened for the activity of alpha-NaGalase. All cell lysates studied contained alpha-NaGalase. The highest expression of the enzyme was determined in DLD-1 cell lines, which have not been studied before. DLD-1 cells grew rapidly, and in a larger amount, enough to study, so this type of cancer cells was chosen for further investigations.

### 2.1. Isolation and Purification of Alpha-NaGalase from Cell Lysates

**Figure 1:**
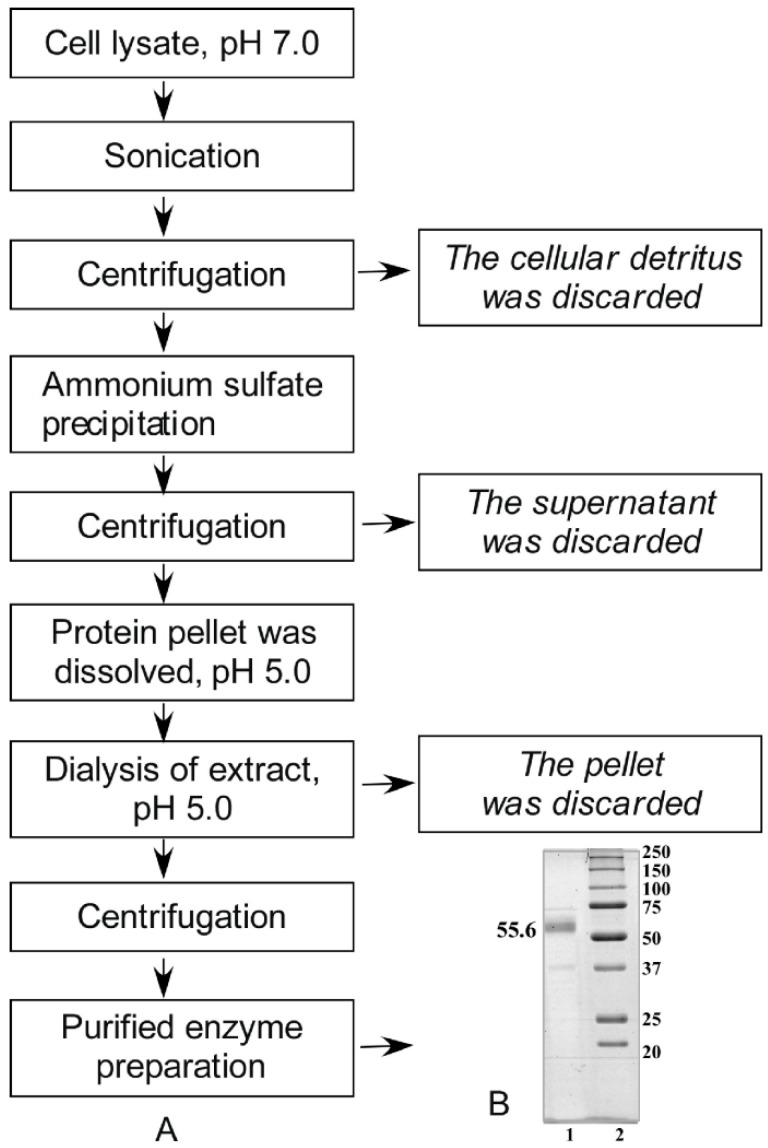
Purification of alpha-NaGalase from lysate of cancer cells DLD-1: (**А**) Scheme of enzyme purification; (**B**) 12% SDS-PAGE of purified alpha-NaGalase (1), molecular mass markers (2).

Alpha-NaGalase was isolated and purified from the biomass of these cells in accordance with procedures shown on Figure 1A.

The purification quality was controlled by SDS-PAGE-electrophoresis (Figure 1B). A large number of proteins was present in the cell lysate. Most proteins were precipitated in citrate buffer, pH 5.0. We cannot determine the activity of alpha-NaGalase in the lysate at pH 7.0, EDTA and phosphate ions under conditions of strong dilution. After final stage of treatment we obtained 6 mL of enzyme preparation with 0.63 mg of protein mL^−1^ and specific activity = 0.033 μmol mg^−1^ min^−1^ in 0.05 M sodium citrate buffer, pH 5.0. According to the electrophoresis data high purified preparation of enzyme has major bond of 55.6 kDa.

### 2.2. Biochemical and Enzyme Properties of Alpha-NaGalase from Cell Lysates

We studied the biochemical characteristic of alpha-NaGalase, isolated from human colon carcinoma cell lines DLD-1. This is necessary for further studies involving the enzyme.

#### 2.2.1. Determination of pH Optimum of the Alpha-NaGalase

**Figure 2:**
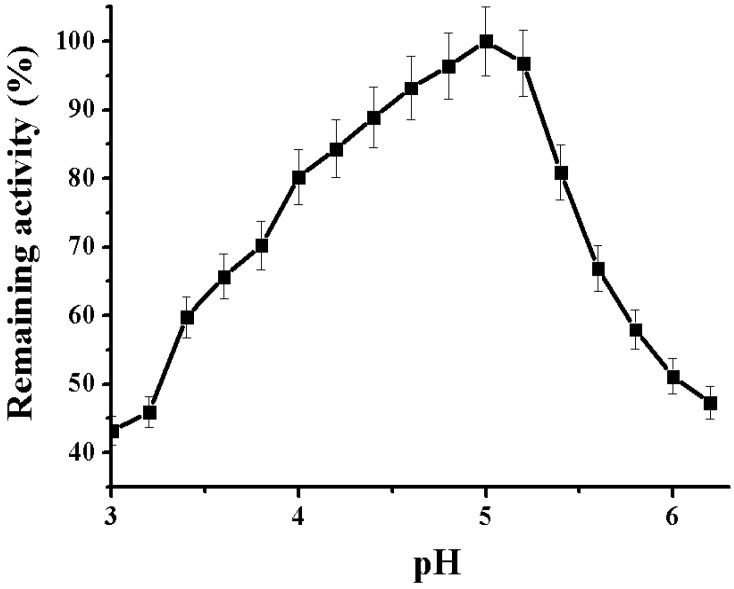
pH-Dependence of alpha-NaGalase activity of DLD-1 human colon carcinoma cells, 0.05 M sodium citrate buffer, 37 °C.

Activity assays in a range of pH values 3.0–6.2 indicated that the enzyme is more than 50% active between pH 4.0 and 5.5 and has an optimum at pH 5.0. However, at pH ≥ 6, the enzyme still retains almost 40% of activity (Figure 2).

This feature of the enzyme from tumor cells is considered a pre-premise for the functioning of the enzyme in the bloodstream at neutral pH [34,35]. The more acidic pH optimum (pH 4.3 and pH 3.5) of action is characteristic for the lyzosomal human liver alpha-NaGalases of GH27 family, especially, of human and chicken liver, respectively [8,10]. Bacterial alpha-NaGalases of GH36, 109 and 129 are active at pH 6.8–7.5 [16,17,18,22].

#### 2.2.2. Effect of Temperature on the Alpha-NaGalase

Alpha-NaGalase of DLD-1 human colon carcinoma cells act in wide range of temperatures.

**Figure 3:**
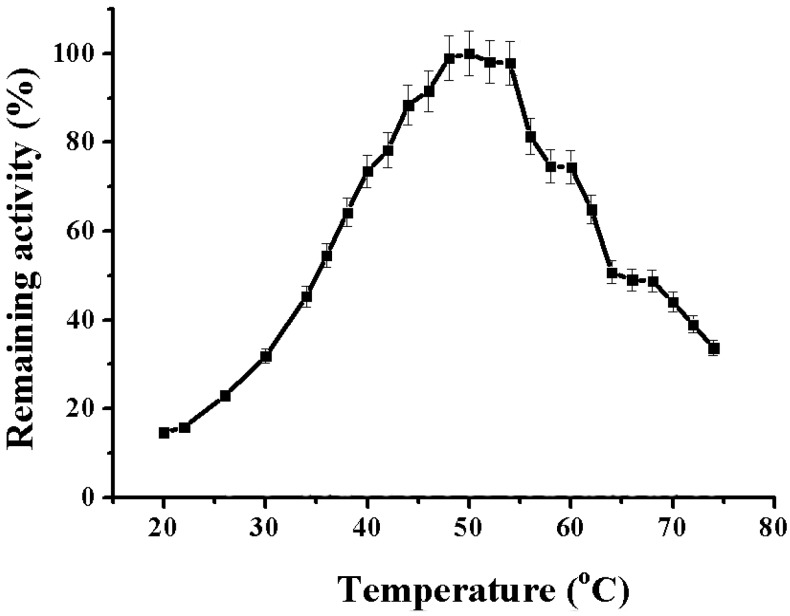
Effect of temperature on the alpha-NaGalase activity of DLD-1 human colon carcinoma cells, 0.05 M sodium citrate buffer, pH 5.0.

When the assay temperature was varied, optimal alpha-NaGalase activity was found at 50 °C, while the enzyme was 50% active at a more physiological temperature of 37 °C (Figure 3). Alpha-NaGalase was completely inactive at temperatures below 10 °C.

**Figure 4:**
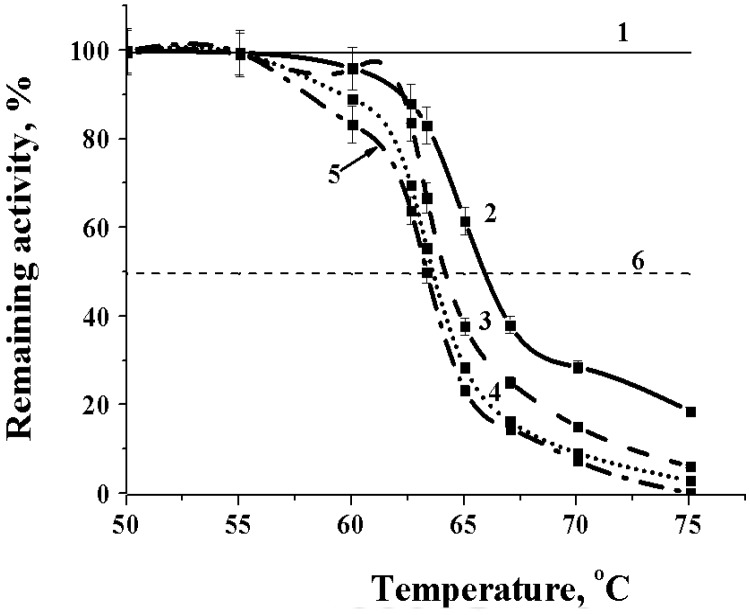
Temperature stability of alpha-NaGalase of DLD-1 human colon carcinoma cells at different exposition time; 1 and 6 correspond to 100% activity and 50% activity of the enzyme, respectively, 0.05 M sodium citrate buffer, pH 5.0; graph 2, 3, 4, 5 correspond to 15, 30, 45 and 60 min of exposition, respectively.

It was shown that the enzyme is quite thermostable. Dependences of remaining activity of the enzyme at different time of exposition are shown on Figure 4.

The alpha-NaGalase of DLD-1 human colon carcinoma cells kept activity up to 75 °C during 60 min exposition. At optimal temperature for catalysis 45 °C the enzyme showed a half-life of 60 min (Figure 4). Furthermore, the purified preparation of the enzyme can be stored frozen for a month and retains 100% activity after thawing.

#### 2.2.3. Effect of Substrate Concentrations on Reaction Rate of Alpha-NaGalase of DLD-1 Human Colon Carcinoma Cells

**Figure 5:**
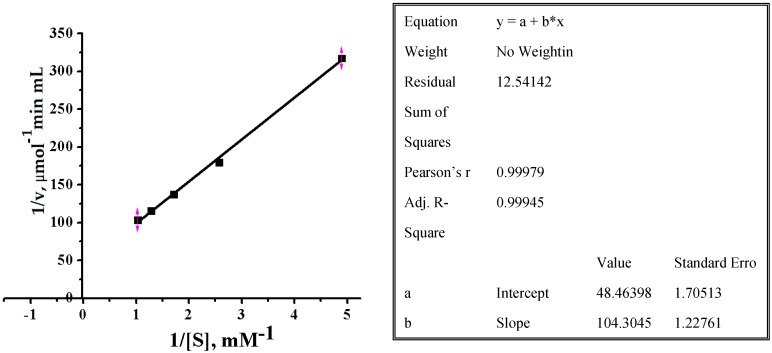
Dependence of *p*-Nitrophenyl-*N*-acetyl-α-d-galactosaminide hydrolysis rate on the concentration catalyzing by alpha-NaGalase of DLD-1 cancer cells in double reciprocal Lineweaver–Burk coordinates, 0.05 M sodium citrate buffer, pH 5.0, 37 °C. The inset table shows the results of liner fitting with using the computer program “Orijin 8.0”.

To determine Michaelis-Menten constant (*K*_m_) and maximal rate of reaction (*V*_max_) for pNPNAGal the substrate concentration was varied between 0.3 and 2.0 mM. Dependence of pNPNAGal hydrolysis rate on the concentration catalyzing by alpha-NaGalase of DLD-1 cancer cells in double reciprocal Lineweaver–Burk coordinates are shown on Figure 5.

Variables *x* and *y* of the Equation in inset table (Figure 5) are reciprocal of a substrate concentration (1/*s*, mM^−1^) and initial velocity of hydrolysis (1/*v*, μmol^−1^ min mL), respectively:

1/*v* = 1/*V*_max_ + *K_m_*/V_max_∙1/*s* (1)

Coefficients *a* (Intercept) and *b* (Slope) of linear fitting are corresponded to *K_m_*/V_max_ and 1/*V*_max_, of Equation (1), respectively. Values of *K*_m_ and *V*_max_ and other parameters for commercial pNPNAGal at 37 °C and pH 5.2 were calculated and summarized in Table 1.

Thus, based on the above, we can conclude that the biochemical properties of the investigated enzyme are similar to the enzyme from cancer cells described earlier [32,33,34,35]. The values of the enzymatic reaction parameters were obtained at the first and were not discussed for mammalian tumor cell alpha-NaGalases [34,35]. The *V*_max_ are significantly lower than for the enzymes of fungi of GH27 family [14,15], as well as bacterial enzymes from the related GH36 family [15,17]. The rate of hydrolysis for commercial glycoside is low, probably because the substrate is not native. The ability to cleave the chromogenic glycoside is an indirect confirmation that the alpha-NaGalase studied is a retaining exo-glycosidase [18].

#### 2.2.4. Effect of NaCl and NaN_3_ on the Alpha-NaGalase

**Figure 6:**
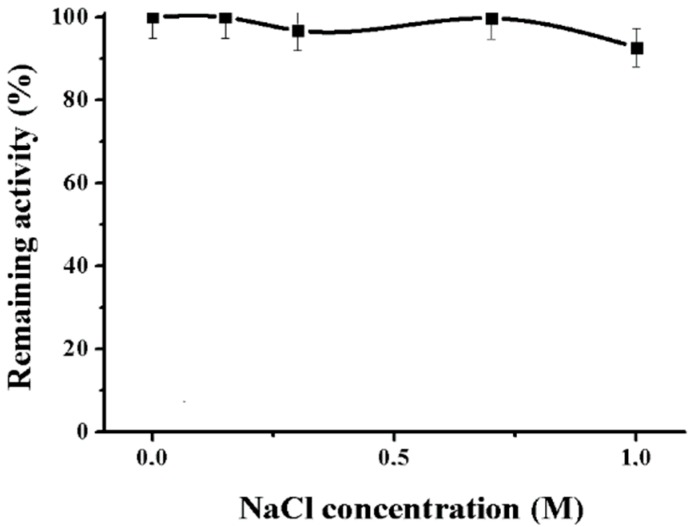
Effect of NaCl on the alpha-NaGalase of DLD-1 human colon carcinoma cells.

It was found that NaCl did not affect the activity of the enzyme (Figure 6).

It is known that the resistance to high concentrations of salt is a characteristic feature of enzymes from marine organisms and human bacterial pathogens. High ionic strength is characteristic of the natural habitat of host organisms, for instance, bloodstream or seawater [18,23].

Sodium-azide is used for conservation of the enzyme preparations for elongated keeping. This agent reduces the activity of the cancer alpha-NaGalase (Table 2). Terefore it should not be used as a conservative agent in this enzyme preparation.

Sodium azide is well known true inhibitor of metallo-enzymes [44]. Alpha-NaGalase GH109 family from marine bacterium *Arenibacter latericius* KMM 426^T^ and α-galactosidase GH36 family from marine bacterium *Pseudoaleromonas* sp. KMM 706 retained 100% activity for a long time at 4 °C and for a week at 20 °C in the presence of 0.1% sodium azide [45,46]. Sodium azide was used as external nucleophile in chemical rescue experiments to probe the catalytic acid/base and the catalytic nucleophile residues of the retaining glycoside hydrolases [47]. However, there are only several reports of azide anions inhibiting the enzyme activity. Sodium azide inhibit *Bacillus licheniformis* 1,3;1,4-α-glucanase [48] and two classes of chitin-degrading enzymes from the marine bacterium *Vibrio harveyi* with distinct modes of action [49]. As strong nucleophile azide anions may bind not only to the carboxyl groups of catalytic sites, but also to the other sites of active center of a glycoside hydrolase [49].

### 2.3. Effect of Fucoidan on the Activity of Alpha-NaGalase

**Figure 7:**
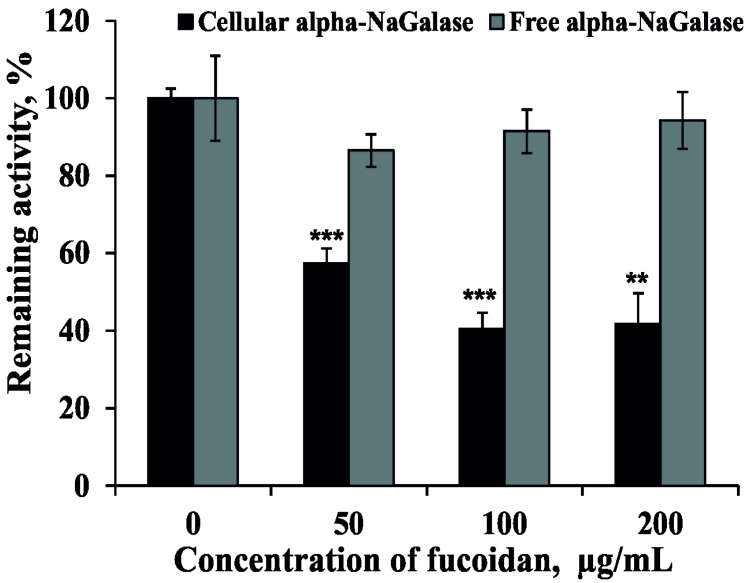
Effect of fucoidan from brown alga *Fucus evanescence* on the production of alpha-NaGalase in colon cancer cells DLD-1 and on the activity of the free alpha-NaGalase, 0.05 M sodium citrate buffer, pH 5.0, the enzyme with fucoidan was incubated for 30 min at 20 °C. Data are shown as means ± standard deviation (SD) of values from three independent experiments. The asterisk (*) indicates a significant decrease in activity of cellular alpha-NaGalase of DLD-1 cells treated with fucoidan compared with the non-treated cells (** *p* < 0.01, *** *p* < 0.001.).

Previously we have isolated and determined the structural characteristics of the fucoidan from brown alga *F. evanescens*. It was shown that fucoidan from *F. evanescens* contained alternating (1 → 3)- and (1 → 4)-α-l-fucose residues sulfated and partially acetylated at C2 position. Additional sulfates occupy C4 position in some 3-linked α-l-fucose residues [50,51]. The effect of fucoidan from the brown alga *Fucus evanescence* on the activity of alpha-NaGalase in human colon carcinoma DLD-1 cells and on the biosynthesis of this enzyme were investigated. It was shown that fucoidan did not inhibit free alpha-NaGalase, however, it reduced the expression of the enzyme in the cancer cells at IC_50_ = 73 ± 4 μg mL^−1^ (Figure 7).

It is known, that anti-cancer and anti-metastatic activities of plant polysaccharides are related with ability directly or indirectly reduce the activity of macrophages [52,53]. Polysaccharides isolated from the algae previously have been reported to enhance the phagocytic and secretory activity of macrophages and induce the production ROS, NO and cytokines (TNF-a, IL-1 and IL-6) [54]. In addition, polysaccharides isolated from blue-green algae, *Spirulina platensis*, exhibited anti-tumor and anti-metastatic activity [54]. Thus, it is likely that algal polysaccharides modulate macrophage immune function. It has been shown, that ionogenic polysaccharides from an edible brown alga, *Hijikia fusiforme* (Hijiki), have a possible enhancing activity for macrophage-dependent suppression against tumor cell growth [55]. Finally, more recently, it has been shown that the immunomodulating effect is one of the possible mechanisms of the protective action of fucoidan against carcinogenesis of the breast [56].

## 3. Materials and Methods

### 3.1. Materials and Reagents

Roswell Park Memorial Institute Medium (RPMI 1640), phosphate buffered saline (PBS), l-glutamine, penicillin-streptomycin solution, trypsine and fetal bovine serum (FBS), sodium hydrocarbonate (NaHCO_3_) and agar were purchased from BioloT (Bolshoy Sampsonievsky Ave, St. Petersburg, Russia), *p*-nitrophenyl-*N*-acetyl-α-d-galactosaminide (p-NPNAGal) were purchased from Sigma-Aldrich (St. Louis, MO, USA), recombinant protein markers SDS-PAGE-electrophoresis–from BioRad (1000 Alfred Nobel Drive, Hercules, CA, USA).

### 3.2. Brown Alga and Fucoidan Isolation

Brown alga *Fucus evanescens* C. Agardh (Fucales/Fucaceae) was collected by hand at littoral zone from natural habitats of Kunashir Island (Pacific Coast) in August, 2012. Thallus of fresh algal biomass was washed thoroughly with tap water, air-dried, and treated sequentially with ethanol, acetone, and chloroform. Samples of defatted, air-dried and powdered algal fronds were extracted twice with 0.1 M HCl for 2 h at 60 °C. The extracts were collected by centrifugation, combined, dialyzed, concentrated, and precipitated with four volumes of 96% ethanol. The precipitates were washed with 96% ethanol and air-dried. This powder of polysaccharide extract was stored at −80 °C before its using for isolation and purification of fucoidan. The fucoidan was isolated from brown alga *F. evanescens*, purified and structural characterized as described in our previous studies [50,51].

### 3.3. Cell Cultured and Treated with Fucoidan

Human colon carcinoma DLD-1 cells (ATCC^®^ no. CCL-221) were obtained from the American Type Culture Collection (Manassas, VA, USA). DLD-1 cells were cultured in RPMI-1640 medium. Culture media were supplemented with 10% FBS and 1% penicillin-streptomycin solution. The cell cultures were maintained at 37 °C in humidified atmosphere containing 5% CO_2_.

#### 3.3.1. Preparation of Cell Lysate

Every 3–4 days DLD-1 cells were rinsed in phosphate buffered PBS, detached from the tissue culture flask by 1X trypsin/EDTA solution, harvested with RPMI-1640 medium and centrifuge at 500 rpm for 3 min. The culture media was discarding and cells pellet was resuspended in EDTA/Tris solution and frozen at −80 °C.

#### 3.3.2. Fucoidan’s Treatment of Cells

DLD-1 cells (5 × 10^5^ cells/dish) were seeded in 60 mm dishes. After 24 h, the cells were treated with a medium containing different concentrations of the fucoidan from *F. evanescens* (50, 100, 200 μg mL^−1^). After 24 h, the cells were harvested as described in Section 3.3.1. “Preparation of cells lysate”. After each procedure of treating cells with fucoidan, cell lysates were prepared, the enzyme was extracted and its specific activity was determined, as described below in the Section 3.4*.*

### 3.4. Isolation and Purification of α-N-Acetylgalactosaminidase from Cell Lysates

Frozen lysates of cancer cells in the 15 mM Tris buffer, pH 7.0 containing 0.02% EDTA, defrosted and sonicated using an ultrasonic homogenizer Bandelin Sonopuls (Bandelin electronic GmbH & Co., KG Heinrichstraße 3–4 12,207, Berlin, Germany) 10 times during 1 min with a break of 20 s. To remove the cellular detritus, the cell homogenate was centrifuged at 4 °C, 10,000 rpm, 30 min. The supernatant proteins were precipitated with 70% ammonium sulfate, kept overnight at 4 °C to form a pellet. The protein pellet was collected by centrifugation (4 °C, 10,000 rev/min, 30 min) and dissolved in 0.05 M sodium citrate buffer, pH 5.0. The extract was dialyzed with dialysis sacks Sigma-Aldrich (St. Louis, MO, USA) against the same buffer, centrifuged to separate the insoluble precipitate. The supernatant was used in further work as purified enzyme. The purification quality was controlled by 12% Laemmli-SDS-PAGE-electrophoresis [57].

### 3.5. Biochemical and Enzyme Properties of α-N-Acetylgalactosaminidase from Cell Lysates

#### 3.5.1. The Molecular Weight

The molecular weight of alpha-NaGalase was evaluated by 12% SDS-PAGE-electrophoresis using recombinant protein markers 250, 150, 100, 75, 50, 37, 25, 20 kDa.

#### 3.5.2. Enzyme Assay

To assay the alpha-NaGalase activity, the following standard procedure was used: 0.05 mL of cell extract and 0.1 mL of substrate pNPNAGal in 0.05 M Na citrate buffer, pH 5.0, were placed in cell of 96-well plate and incubated at 37 °C for 30 min. The reaction was stopped by the addition of 0.15 mL of a solution of 1 M Na_2_CO_3_. Absorbance was measured at 400 nm on PowerWave XS microplate spectrophotometer (BioTek Instruments, Highland Park, Winooski, VT, USA). Results were read with a computer program “Gen5” and treated with “ExCel” computer program. The unit of activity (U) was defined as the amount of the enzyme catalyzing the formation of 1 μmol of p-nitrophenol (ε_400_ = 18,300 M^−^^1^ cm^−^^1^) per 1 min under the conditions indicated. Specific activity (A) was calculated as the enzyme activity (U) per 1 mg of protein. All calculations were based on reactions with consumption of 10% of the chromogenic substrate. The protein concentrations were estimated by the Bradford method [58]. Remaining activity (%) was calculated with formula:

Remaining activity (%) = A/A_0_ × 100, (2)

where A_0_ is the specific alpha-NaGalase activity in the absence of any influence factors, at 0.05 M Na citrate buffer, pH 5.0.

#### 3.5.3. pH Optimum of the Alpha-NaGalase

For determination of pH optimum, the mixture contained 0.025 mL of enzyme solution (after the last step of purification), 0.025 mL of 0.1 M Na citrate of buffer pH 3.0–6.2 and 0.1 mL of the substrate (1 mg/mL of H_2_O). The enzyme activity was determined at 37 °C after 30 min of incubation as described above.

#### 3.5.4. Effect of Temperature on the Alpha-NaGalase

To study an effect of temperature on the activity of NaGalase DLD-1 0.05 mL of the enzyme solution in 0.05 M sodium citrate buffer pH 5.0, placed in a warmed cryo-tube, heated in a thermostatic shakier (BioRad, 1000 Alfred Nobel Drive, Hercules, CA 94547, USA) at 20 to 74 °C for 1 min, then 0.1 mL of in advance heated p-nitrophenyl-α-*N*-acetyl-galactosaminide (2.93 mM) in a solution of the same buffer was added. The enzyme activity was determined at each temperature as described above. For the alpha-NaGalase DLD-1 temperature stability study 0.05 mL of the enzyme solution was heated for 15, 30, 45 and 60 min in the temperature range from 50 to 75 °C. The samples were cooled and their activity was determined as described above. From the dependences of the residual activity (A/A_0_) on the incubation time at different temperatures (T °K), the thermodynamic parameters of the thermal inactivation of alpha-NaGalase were determined.

#### 3.5.5. Effect of NaCl and NaN_3_ on the Alpha-NaGalase

To study an effect of NaCl on the activity of alpha-NaGalase DLD-1 0.05 mL of NaCl solution at different concentration were added to 0.05 mL of substrate solution and 0.05 mL of enzyme. The mixtures were incubated for 30 min at 37 °С. The enzyme activity was determined as described above.

To study an effect of NaN_3_ on the activity of alpha-NaGalase DLD-1 0.05 mL of the enzyme was mixed with 0.005 and 0.015 mL of an aqueous solution of 1% NaN_3_ to obtain a concentration of 0.1 and 0.3% of the agent in the test mixture. The mixtures were held for 30 min, then 0.095 mL and 0.085 mL of substrate buffer solution (5 mM), respectively, were incubated for 30 min at 37 °C, the reaction was stopped by the addition of 0.15 mL of Na_2_CO_3_. The activity was determined as described above. Mixtures of 0.05 mL of enzyme, 0.005 and 0.015 mL of water, as well as 0.095 mL and 0.085 mL of working buffer were used as a control.

#### 3.5.6. Determination of *K*_m_ and *V*_max_ Values

For determination of *K*_m_ and *V*_max_ values of alpha-NaGalase the substrate solution in different concentration was added to 0.05 mL of enzyme solution (0.63 mg mL^-1^) and incubated at 37 °С for 60 min. The final substrate concentrations were 0.39, 0.78, 1.17, 1.56, 1.95 mM in the incubation mixtures. The reactions were stopped by the addition of 0.15 mL of Na_2_CO_3_. The activity was determined as described above. The Michaelis-Menthen constant *K*_m_ and *V*_max_ were determined from the coefficients of linear regression of the Lineweaver-Burke plot (double-reciprocal).

### 3.6. Effect of Fucoidan on Alpha-NaGalase

To elucidate the effect of fucoidan on alpha-NaGalase of DLD-1 cancer cells 0.025 mL of water solution of fucoidan from brown alga *Fucus evanescence* were added to 0.025 mL of the enzyme solution. Mixtures were incubated for 30 min, then 0.1 mL of substrate solution in sodium citrate buffer, pH 5.0, were added and incubated for 60 min at 37 °С. The enzyme activity was determined as described above. The fucoidan concentrations were 50, 100, 200 μg/mL in incubation mixture. Mixture of 0.025 mL of enzyme and 0.025 mL of water was used as a control of 100% activity.

## 4. Conclusions

In the present work, we isolated and purified the almost homogeneous preparations of the alpha-NaGalase from colon cancer cell line DLD-1. The enzyme has been shown earlier deglycosylates the GcMAF and inhibits macrophage activity acting as an immunosuppressor. Physicochemical and catalytic properties of the enzyme were characterized using the standard chromogenic glycoside. By its biochemical properties, the enzyme was similar to the known enzymes from cancer cells. Previously the enzyme has been shown to deglycosylate the GcMAF and inhibits macrophage activity acting as an immunosuppressor. Sulfated polysaccharide fucoidan from brown alga *F. evanescens* possessing with wide range of biological activities including immunomodulating activity did not inhibit free alpha-NaGalase but reduced the expression of the enzyme by DLD-1 cell lines. We have assumed that the capacity to reduce the expression of an aggressive enzyme is one from side of immunomodulating properties of the polysaccharide. However, further in vitro and in vivo studies are needed to investigate the molecular mechanism of fucoidans inhibitory activity on alpha-NaGalase expression. Overall, alpha-NaGalase from colon cancer DLD-1 cell lines and from other cancer cell lines potentially may be used for searching of inhibitors from different marine hydrobionts, which can serve a base for creation of novel anticancer drugs.

## Figures and Tables

**Table 1:** The main characteristics of alpha-NaGalase of DLD-1.

**Molecular Mass, kDa**		55.6
**Optimum pH range**		4.6–5.0
**Temperature stability**	Optimum temperature range	50 °C
	Start of inactivation	62 °C
	50% inactivation	63–67 °C
	100% inactivation	75 °C
**Catalytic properties**	*K*_m_, mM	2.15
	*V*_max_, μmol min^−1^ mL^−1^	0.021
	*k*_cat_, min^−1^	1.55
	*k*_cat_/*K*_m_, min^−1^ mM^−1^	0.72

**Table 2:** Effect NaN_3_ on activity of alpha-NaGalase.

NaN_3_, %	Remaining Activity, %
0	100
0.1	85
0.3	67
